# Impaired neutrophil directional chemotactic accuracy in chronic periodontitis patients

**DOI:** 10.1111/jcpe.12326

**Published:** 2015-01-08

**Authors:** Helen M Roberts, Martin R Ling, Robert Insall, Gabriela Kalna, Julia Spengler, Melissa M Grant, Iain LC Chapple

**Affiliations:** Periodontal Research Group and MRC Centre for Immune Regulation, University of BirminghamBirmingham, UK

**Keywords:** chemoattractant, chemotaxis, neutrophil, periodontitis, treatment

## Abstract

**Aim:**

To investigate the chemotactic accuracy of peripheral blood neutrophils from patients with chronic periodontitis compared with matched healthy controls, before and after non-surgical periodontal therapy.

**Material & Methods:**

Neutrophils were isolated from patients and controls (*n* = 18) by density centrifugation. Using the Insall chamber and video microscopy, neutrophils were analysed for directional chemotaxis towards *N*-formyl-methionyl-leucyl-phenylalanine [fMLP (10 nM), or CXCL8 (200 ng/ml)]. Circular statistics were utilized for the analysis of cell movement.

**Results:**

Prior to treatment, neutrophils from patients with chronic periodontitis had significantly reduced speed, velocity and chemotactic accuracy compared to healthy controls for both chemoattractants. Following periodontal treatment, patient neutrophils continued to display reduced speed in response to both chemoattractants. However, velocity and accuracy were normalized for the weak chemoattractant CXCL8 while they remained significantly reduced for fMLP.

**Conclusions:**

Chronic periodontitis is associated with reduced neutrophil chemotaxis, and this is only partially restored by successful treatment. Dysfunctional neutrophil chemotaxis may predispose patients with periodontitis to their disease by increasing tissue transit times, thus exacerbating neutrophil-mediated collateral host tissue damage.

Chronic periodontitis is a disease that is initiated by the emergence of a pathogenic biofilm and characterized by non-resolving inflammation which leads to host-mediated tissue damage and bone loss around the teeth (Grossi et al. 1994). Chronic periodontitis can itself be a risk factor for other inflammatory diseases including type 2 diabetes (Chapple et al. 2013), rheumatoid arthritis (RA) (de Pablo et al. 2009) and cardiovascular diseases (Dietrich et al. 2013). The disease is characterized by a strong neutrophil tissue infiltrate (Van Dyke 2009) and tissue damage progresses as a result of abnormal host inflammatory-immune processes, eventually resulting in bone resorption and a receding gingival epithelial attachment (Graves & Cochran 2003).

The oral tissues are constantly exposed to foreign and potentially harmful microorganisms, and in order to combat potential infections in this vulnerable area, immune surveillance involves leucocyte infiltration into the tissues from the blood stream in response to endogenous and exogenous chemoattractants (Gamonal et al. 2001). Immune cells, including neutrophils, are recruited to the site of infection by chemokines such as CXCL8 (Interleukin-8) and CCL3 (macrophage inhibitory protein-1alpha-MIP1α) and other inflammatory stimuli including the bacteria-derived *N*-formyl-methionyl-leucyl-phenylalanine (fMLP). The ability of neutrophils to efficiently reach the site of inflammation is crucial in order to eliminate potentially pathogenic agents, whilst minimizing collateral host tissue damage. Pathogen-associated molecular patterns (PAMPs), damage-associated molecular patterns (DAMPs) and other molecules with chemoattractive properties, which include complement proteins such as C5a, and eicosanoids (leukotriene B4) and platelet activating factor (PAF), have different potencies, forming a chemical hierarchy that serves to recruit neutrophils to the source of inflammation. Those that elicit the strongest migration are molecules that emerge from the inflammation/infection source, including bacterial products such as fMLP.

Chemotaxis, the directional movement of cells in response to chemical gradients, is a highly conserved process occurring in a diverse number of organisms; in particular, there are strong similarities between cell movement in the unicellular *Dictyostelium discoideum* and neutrophils, both of which are able to navigate along shallow chemoattractant gradients (Van Haastert & Devreotes 2004). In the case of neutrophils, chemotaxis allows the cell to reach the infected/colonized area, in order to effect phagocytosis and subsequent destruction of the microorganisms by reactive oxygen species (ROS) and proteolytic enzymes, within the safe confines of the phagolysosome (Cooper et al. 2013). A number of interacting processes must occur for effective, coordinated cell movement, including recognition of the chemoattractant, internal signalling to reach the cells motility centre and gradient detection to influence movement in a persistent direction (Kolaczkowska & Kubes 2013). Chemoattractant binding induces polymerization of F-actin, the formation of new pseudopods at the leading edge and retraction at the posterior edge of the cell (Andrew & Insall 2007). In the absence of chemoattractants, these protrusions occur randomly at all edges of the cell. However, when a chemoattractant is detected, the protrusions are directed towards the source of the chemoattractant, determining the direction of migration (Andrew & Insall 2007).

To recognize the chemoattractant signal, neutrophils employ a number of receptors that are members of the transmembrane G-protein-coupled receptor (GPCR) family, activation of which triggers various signalling cascades that enable movement in a direction-specific manner. Both exogenous agents, such as bacteria-derived products, and endogenous factors, such as chemokines, activate respective GPCRs resulting in internalization, chemokine degradation and receptor recycling back to the cell membrane (Samanta et al. 1990). Downstream signalling (Fig. 1) results in the activation of the cytoskeleton in order for the cell to move. Receptor–ligand binding of chemoattractants results in the activation of phosphatidylinositol 3′-kinases (PI3Ks), protein kinases C (PKCs), tyrosine kinases, mitogen-activated protein kinases (MAPKs) and GTP binding proteins (Worthen et al. 1994). GPCR stimulation also induces intracellular calcium release via the inositol triphosphate and ryanodine receptors, which has been shown to be important for cellular chemotaxis (Berridge et al. 2003). Another receptor, activating intracellular calcium stores in neutrophils, stimulated by fMLP is the CD38 membrane glycoprotein (Partida-Sanchez et al. 2001). Intracellular calcium is released via the ryanodine receptor as a result of CD38 binding (Kurihara et al. 1993), and PI3K catalyses the formation of phosphatidylinositol 3,4,5-triphosphate (PIP_3_) from phosphatidylinositol 4,5-biphosphate (PIP_2_). PIP_3_ acts as a second messenger controlling cell adhesion and cytoskeletal reorganization (Toker & Cantley 1997). In a cell, responding to a chemoattractant PIP_3_ is found at the leading edge of the cell (Bagorda & Parent 2008). Downstream of receptor signalling the MAPK signalling pathway is also activated (Tsai et al. 2013). At the lagging end of the cell, retraction of the cell membrane is mediated by the phosphatase and tensin homologue (PTEN).

**Fig 1:**
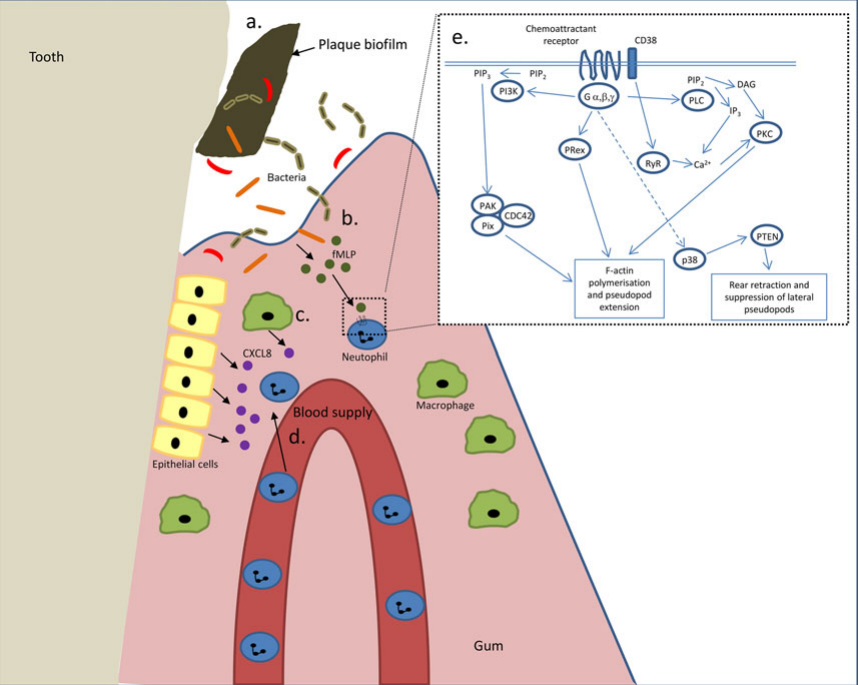
Neutrophil recruitment to inflamed periodontal tissues. (a) The plaque biofilm is formed of diverse species of bacteria. (b) During infection, bacteria and their products penetrate the tissues surrounding the tooth and bacterial degradation products, such as fMLP, are released. fMLP is a potent chemoattractant. (c) After exposure to bacteria, resident macrophages and epithelial cells secrete CXCL8, another potent chemoattractant. (d) CXCL8 and fMLP attract circulating neutrophils, which leave the blood supply and enter the tissues to combat bacterial invasion. (e) Schematic representation of signalling events downstream of chemoattractant–receptor ligation. Upon binding to GPCR G-proteins dissociate and activate various proteins eventually resulting in movement of the cell via actin polymerization. Abbreviations: fMLP, formyl-methionyl-leucyl-phenylalanine; CXCL8, interleukin-8; PIP3, phosphatidylinositol 3,4,5-triphosphate; PIP2, phosphatidylinositol 4,5-biphosphate; PI3K, Phosphatidylinositol 3-kinase; PAK, p21-activated kinase; Prex, phosphatidylinositol-3,4,5-trisphosphate-dependent Rac exchange factor; RyR, ryanodine receptor located on intracellular calcium stores (e.g. endoplasmic reticulum); PLC, phospholipase C; PKC, protein kinase C; PTEN, phosphatase and tensin homologue.

In addition to being a powerful microbicidal weapon employed by neutrophils, reactive oxygen species (ROS) produced by NADPH oxidases are also generated by chemoattractant stimulation and have been shown to play a role in signal transduction of cell movement (Dickinson & Chang 2011). Sakai et al. 2012 demonstrated that ROS produced by NADPH oxidase activity could regulate pseudopod formation and chemotactic migration in neutrophils via actin glutathionylation and polymerization. They also showed that inhibition of NADPH oxidase-dependent ROS formation within healthy neutrophils led to diminished chemotaxis efficiency when exposed to a chemoattractive gradient. Hydrogen peroxide, a membrane-permeable ROS, was able to direct cell movement in a gradient-driven manner (Niethammer et al. 2009), a finding supported by another study in which ROS were found to deactivate PTEN resulting in the build-up of PIP_3_ at the leading edge of the migrating cell, necessary for chemotaxis (Kuiper et al. 2011).

Defective neutrophil chemotaxis features in several diseases including actin dysfunction syndrome, Chediak–Higashi syndrome, Crohn's disease and localized aggressive periodontitis (LAP) (Lakshman & Finn 2001). Although some studies have been published on neutrophil migratory behaviour in periodontitis (Clark et al. 1977, Van Dyke et al. 1980, Daniel et al. 1993), very few have been dedicated to the study of chronic periodontitis. In LAP, previously known as localized juvenile periodontitis (LJP) (Kantarci et al. 2003), a significant number (65–75%) of LAP sufferers have been shown to exhibit defective neutrophil chemotaxis (Lavine et al. 1979, Van Dyke et al. 1980, 1987, Page et al. 1985). Chemotaxis studies using Boyden chambers have reported impairment of neutrophil movement in chronic periodontitis relative to control subjects (Kumar & Prakash 2012). However, there is no information on visualization of chemotaxis in patients with chronic periodontitis; this measures the cell migration path in more detail, recording the direction, speed, velocity and morphology of the cells undergoing chemotaxis (Sackmann et al. 2014).

We have previously shown that peripheral blood neutrophils isolated from patients with chronic periodontitis display both a hyperactive phenotype (i.e. excess ROS production when unstimulated) and a hyperreactive phenotype (i.e. excess ROS production upon stimulation) relative to age- and gender-matched controls (Matthews et al. 2007a,b). Here, in order to expand on these findings and to evaluate neutrophil chemotaxis, we characterize the chemotactic response of peripheral neutrophils from patients with chronic periodontitis, compared with age- and gender-matched controls, prior to and following non-surgical periodontal therapy using advanced time-lapse microscopy techniques developed to study neutrophil movement in shallow chemoattractant gradients.

## Materials and Methods

### Study populations

Thirty-six volunteers were enrolled into this intervention study, including 18 with chronic mild–moderate periodontitis (eight females; 10 males; age mean ± standard deviation 46 ± 7 years) and 18 gender- and age-matched periodontally healthy controls (46 ± 8 years). All volunteers were never smokers and otherwise in good general health, as confirmed by a detailed medical history questionnaire. Chronic periodontitis was defined as the presence of at least two non-adjacent sites per quadrant with probing pocket depths >4 mm, which bled on probing and which demonstrated radiographic bone loss ≥30% of the root length (non-first molar or incisor sites) (Matthews et al. 2007a). Control patients had no evidence of attachment loss, no probing pocket depths >4 mm and whole-mouth bleeding scores <10%. Inclusion criteria were the complete absence of vitamin supplements, no use of anti-inflammatory or antibiotic medication in the previous 3 months, no pregnancy, mouthwash use or special dietary needs (Brock et al. 2004). All volunteers provided written informed consent, and ethical approval for the study was obtained from the West Midlands Research Ethics Committee (number 10/H1208/48). After enrolment, all volunteers were re-appointed for collection of baseline blood samples and clinical measures. Patients received oral hygiene instruction and conventional non-surgical therapy, in the form of scaling and root surface debridement (RSD), performed under local anaesthesia on a quadrant-by-quadrant basis within a maximum of 4 weeks. Patients were recalled 3-months post-therapy to provide a repeat blood sample and clinical measures. A 3-month recall was chosen to allow for initial healing and to reduce the risk of re-infection/disease re-activation (Chapple et al. 2007a,b). Neutrophil isolation and chemotaxis data were obtained for volunteers following treatment along with their matched healthy controls. Clinical data for the patients with periodontitis pre- and post-treatment and for the healthy volunteers is shown in Table 1.

**Table 1:** Age, probing pocket depths, number of sites >4 mm, percentage sites with bleeding on probing, and gingival and plaque indices of patient and healthy control volunteers

	Patients with chronic periodontitis		Healthy controls (*n* = 18)
	Pre-treatment (*n* = 18)	Post-treatment (*n* = 16)	
Probing pocket depths (mean ± SD)	3.0 ± 0.9 (*p* < 0.001)*	2.2 ± 0.6 (*p* < 0.001)#	1.6 ± 0.4
Probing pocket depths >4 mm (median; range)	26.5 (5–91) (*p* < 0.001)*	7.5 (0–52) (*p* < 0.01)#	0 (0–4)
% bleeding on probing (median; range)	41.5 (16–87) (*p* < 0.001)*	14 (3–35) (*p* < 0.001)#	1.5 (0–39)
Gingival index (median; range)	2 (1–3) (*p* < 0.001)*	1 (0–1) (*p* < 0.01)#	1 (0–1)
Plaque index (median; range)	2 (1–3) (*p* < 0.01)*	1 (0–2) (*p* < 0.01)#	1 (0–2)

* *p* values in parenthesis are comparisons with controls.

# *p* values in parenthesis are comparisons with chronic periodontitis before treatment.

### Collection of blood and preparation of neutrophils

Venous blood was collected from the ante-cubital fossa into VacutainerTM lithium heparin (17 IU/ml) tubes, and neutrophils were isolated using Percoll density gradients (GE Healthcare) as previously described (Matthews et al. 2007a). Briefly, two discontinuous gradients, 1.079 and 1.098, were used for neutrophil isolation with concomitant erythrocyte lysis (0.83% NH_4_Cl containing 1% KHCO_3_, 0.04% EDTA and 0.25% BSA). Isolated cells were re-suspended in PBS supplemented with glucose (1 mM) and cations (1 mM MgCl_2_, 1.5 mM CaCl_2_) at 1 × 10^6^ cells/ml. Cell viability, typically >98%, was determined by dye exclusion (trypan blue). Cell purity was determined by cytospin and fluorescence-activated cell sorting (FACS) using CD15 and CD66 neutrophil surface markers.

### Chemotaxis protocol

**Fig 2:**
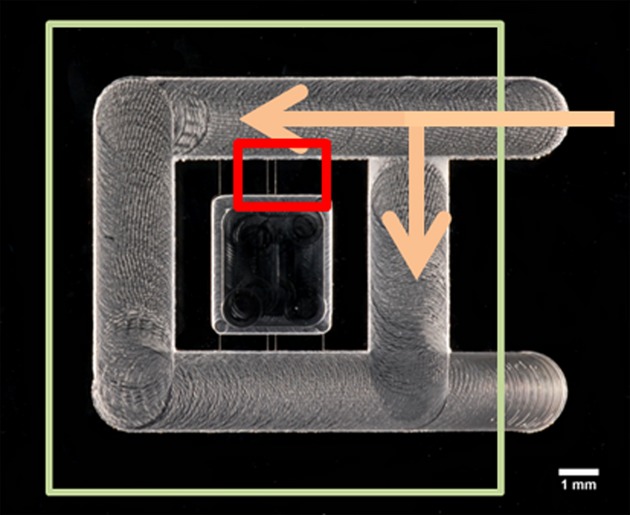
Photograph of the Insall chamber. Large square illustrates position of the coverslip with adhered neutrophils. Arrows show the application of the chemoattractant. Small red rectangle shows the area visualized by video microscopy.

The Insall chamber was used to visualize chemotaxis (Muinonen-Martin et al. 2010). For each sample, isolated neutrophils (400 μl in RPMI, final density 1 × 10^6^/ml) were added to acid washed (0.2 M HCl), dried and blocked (7.5%, BSA 400 μl, Sigma) coverslips (22 mm, VWR International), which were then incubated at room temperature (approximately 23°C) for 30 min. to allow the cells to adhere. The coverslip was then inverted and placed at the top of the chemotaxis chamber ensuring that the chemoattractant loading bays were exposed (Fig. 2). The desired chemoattractant (80 µl, fMLP (10 nM) or CXCL8 (used at 200 ng/ml after assessing a range of concentrations) or control (RPMI media) was injected into the chemoattractant channels. Cell movement was analysed using a Zeiss Primovert microscope (Carl Zeiss Imaging, Thornwood, NY, USA) and Images captured every 30 s for up to 40 frames per condition using a Q Imaging Retiga 2000R camera (Qimaging, Surry, Canada).

### Image analysis

The images generated by video microscopy were processed using Q pro-imaging software (Surrey, Canada) and analysed further using ImageJ 1.45SR software (National Institutes of Health, Bethesda, USA). The manual tracking plug-in (MtrackJ) was employed, and for each set of images, 15 cells were chosen at random and tracked through the frames. The numerical data generated was used to calculate cell speed, cell velocity and chemotactic index (CI) per experiment. The numerical data generated were then used to calculate cell migration, which was defined as follows:

1. Cell speed: the average speed of a cell in any direction over the time course.
2. Cell velocity: the average speed of a cell in its most prominent direction over the time course.
3. Chemotactic Index: this is a measure of the directional accuracy of chemotaxis. It is calculated as a change in the angle of a cell along the Y axis according to the cosine plot (Andrew & Insall 2007).

### Statistical analysis

**Fig 3:**
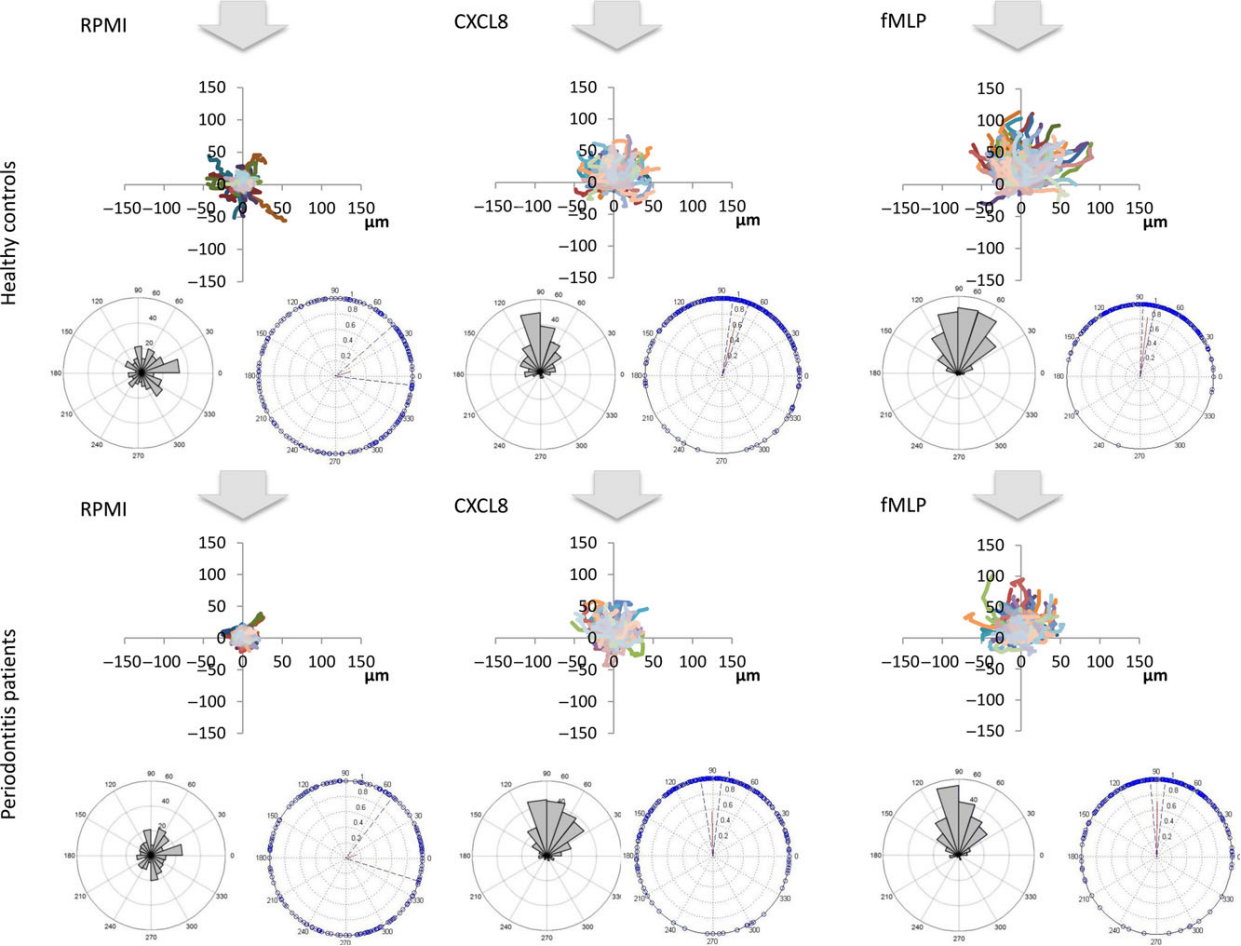
Summary of Pre-treatment results: arrows denote the origin of the chemoattractant, cells should be attracted towards the arrow; spider diagrams show movement of all cells (um) from place of origin; left hand side vector plots show the proportion of cells in each segment and the angle of the segment towards the arrow; right hand side rose plots show the strength of movement and its directionality for the whole cohort of cells, the small red line shows the vector and the bounding dashed lines show the variation within the data. All diagrams are represented at the same scale to aid comparison.

**Fig 4:**
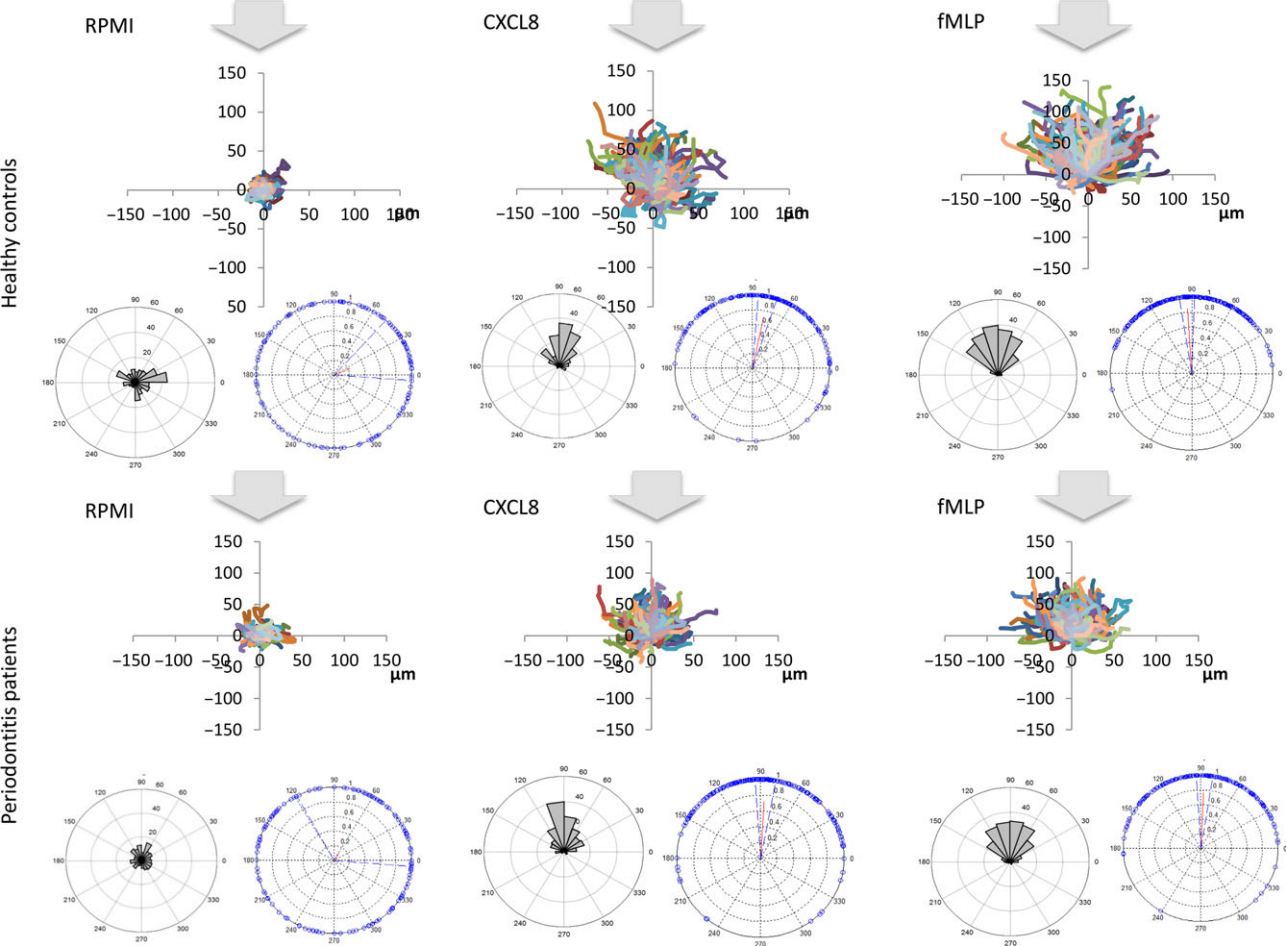
Summary of post-treatment results. The diagrams illustrate three plots per condition, as described in Fig. 3.

XY coordinates of the cells were generated using the Manual tracking Plugin and ImageJ software (Rasband, W.S., ImageJ, U. S. National Institutes of Health, Bethesda, MD, USA) and these were further analysed with the circular statistics (CircStat) toolbox from MATLAB (Mathworks, Natick, MA, USA) software to ascertain the significance of the cells’ movement over the time course. The CircStat toolbox provides statistics for directional data, including the mean direction, known as the resultant vector, the length of which indicates the strength of the direction taken by the cells. Results are represented as circular diagrams. Two representations are shown (Figs 3 and 4): (1) resultant vector plots showing the distribution of the final angle of all cells in the experiment with a vector line showing the mean angle and vector length, illustrating the strength of the movement; and (2) rose plots showing the proportion of cells in each of 18 segments around the circle, the larger the bar the greater proportion of cells that moved in that direction. Data were further summarized in box and whisker plots, and statistical analysis of these was performed by Wilcoxon test using Prism 5.0 software (GraphPad, San Diego, California USA). Volunteer age was compared by paired *t*-test and probing pocket depths by repeated measures anova followed by Tukey–Kramer multiple comparisons test. The number of probing pocket depths >4 mm, percentage bleeding on probing, and gingival and plaque indices were compared using Friedman test followed by Dunn's multiple comparisons test.

## Results

Clinical findings for patients and their matched controls, pre- and post-treatment, are shown in Table 1. Figures 3 and 4 depict summaries of all the data collected pre- or post treatment, respectively. Each dataset comprises three graphs: the top image is a “spider plot” of individual cell movement tracks towards the “12.00 o'clock” position; the lower left image is a rose plot that clusters groups of cells according to their direction of movement; and the lower right image is a vector plot that indicates the strength and angle of movement. It is clear to see the difference in the strength of the two chemoattractants CXCL8 and fMLP, with fMLP producing the strongest response evidenced by longer cell tracks in the spider diagrams. The control-treated cells (RPMI), as anticipated, show very little movement and no obvious directionality of movement.

**Fig 5:**
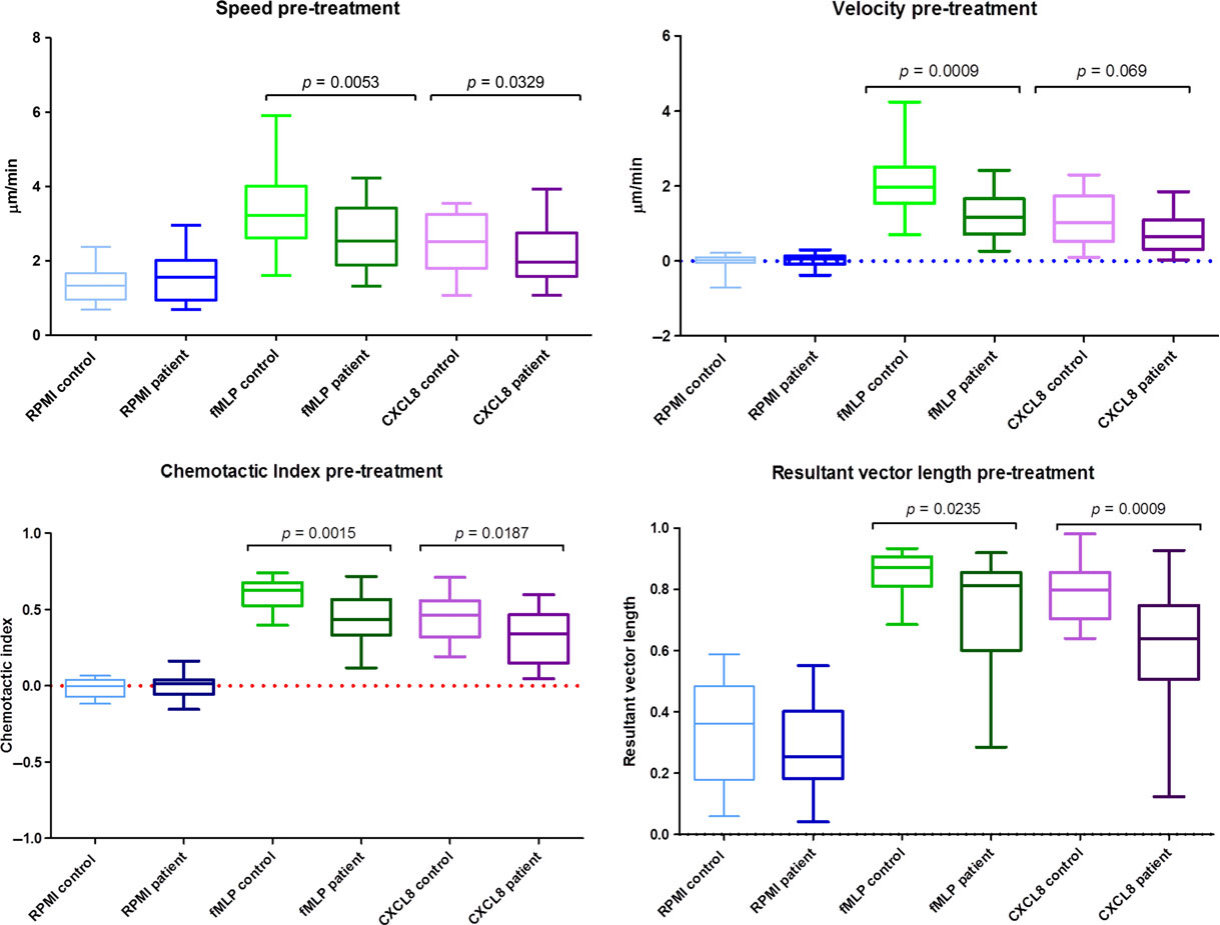
Analysis of pre-treatment results: extracted values for each individual's speed, velocity, chemotactic index and resultant vector length were analysed for statistical difference (Wilcoxon test). The midline of each box represents median, bounding box the 25th and 75th percentiles and the whiskers the extremities of the data sets.

**Fig 6:**
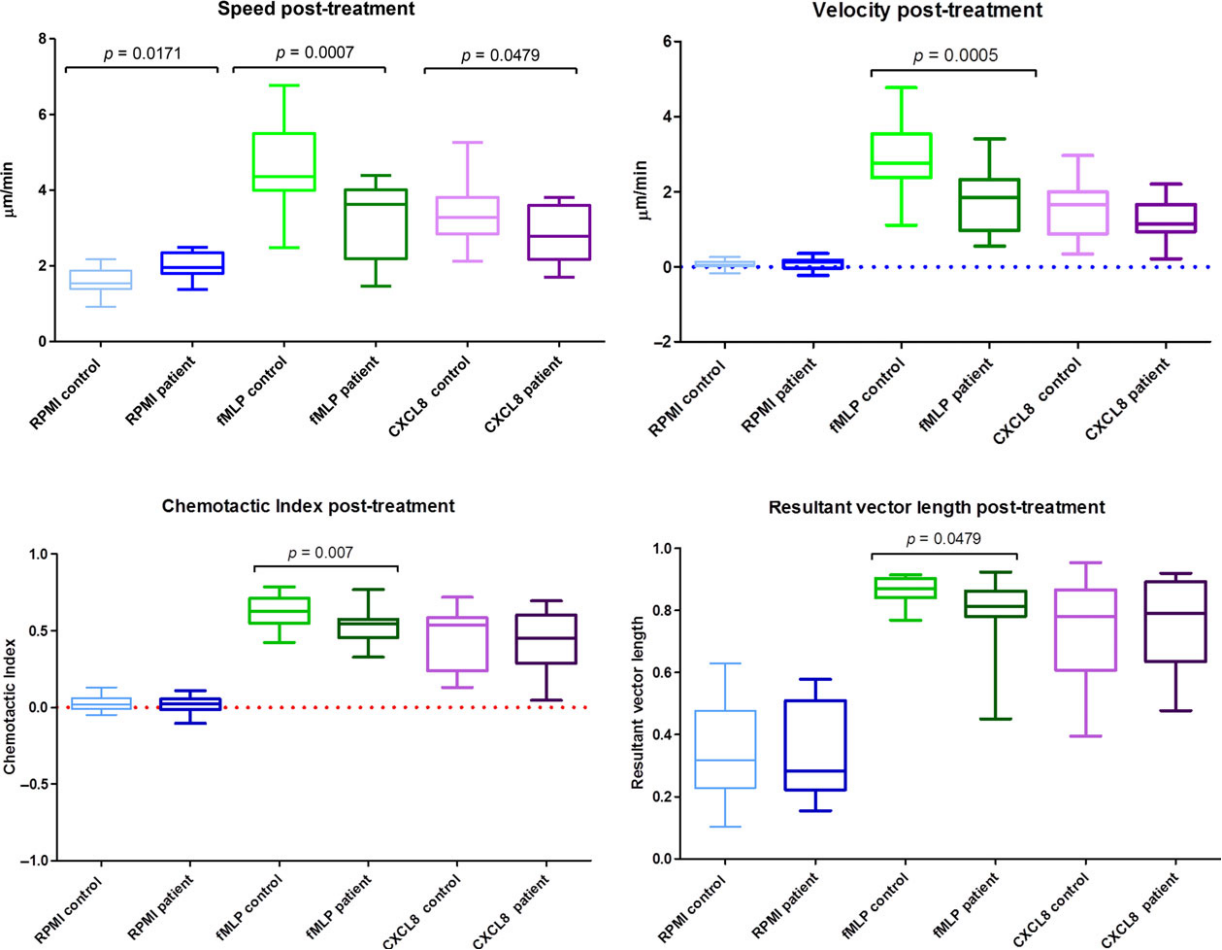
Analysis of post-treatment results: as described in Fig. 5.

Statistical analyses of these data (Figs 5 and 6) demonstrate that before treatment, neutrophils from patients with periodontitis have significantly lower speed, velocity and directional accuracy (chemotactic index and resultant vector length) than neutrophils from healthy controls for both chemoattractants, CXCL8 and fMLP. Following treatment, they still display significantly reduced speed, velocity and accuracy than neutrophils from healthy control volunteers for fMLP; however, with the exception of speed, the neutrophils from patients with periodontitis were not significantly different in their response to CXCL8 following therapy, in comparison to neutrophils from healthy volunteers. Patient and control pre- and post-treatment results were analysed separately because of the high inter-individual variation that arises when neutrophils are analysed on different days. Therefore, patient and control cells were analysed synchronously at both baseline and then again simultaneously following therapy, but no attempt was made to compare patients’ cells pre- and post-treatment (or controls).

## Discussion

This study has demonstrated for the first time that neutrophils from patients with chronic periodontitis exhibit reduced chemotactic accuracy compared to gender- and age-matched controls. Patient neutrophils were less responsive to the widely used chemoattractants fMLP and CXCL8 with regard to chemokinesis and chemotaxis when compared to respective controls.

Direct visualization chambers, including the Zigmond, Dunn and the Insall chambers (the latter was used in this study) allow cells to be observed migrating using time-lapse video microscopy in real time (Wells 2000). Bridge chambers provide a visualization platform for observing the behaviour of cells between two wells. These chambers provide gradients for the cells to accelerate towards rather than exposure to absolute concentrations alone. The majority of studies examining the defects in neutrophil chemotaxis over the last 30 years used the Boyden chamber (and its derivatives) to study chemotaxis (Clark et al. 1977, Van Dyke et al. 1980, 1987, Offenbacher et al. 1987, Daniel et al. 1993, Yagi et al. 2009). Other studies (Henry et al. 1984) employed the checkerboard assay described by Zigmond & Hirsch (1973). The use of the Insall chamber has recently expanded (Phillips & Gomer 2012, Choi et al. 2013, Herlihy et al. 2013a,b, Kaul et al. 2013) and opened up new research questions, which informed the present study. Here, we report on speed, velocity and chemotactic accuracy. Chemotactic accuracy has been expressed as both chemotactic index (Sapey et al. 2011) and as resultant vector analysis (Andrew & Insall 2007); however, here, we have demonstrated that these two factors are interchangeable (Fig. S1, Bland-Altman analysis).

Although there are few studies examining chemotaxis in chronic periodontitis (Kumar & Prakash 2012), there is a wealth of data concerning chemotaxis in patients with localized aggressive periodontitis (LAP). It is important to note that LAP is distinctly different from chronic periodontitis as it occurs in otherwise systemically healthy adolescents; the bacteria that colonize the oral tissues in these individuals are different in composition to chronic periodontitis; and there is a strong genetic pre-disposition to the disease (Fu et al. 2002, Nibali et al. 2008). Defective LAP neutrophils, however, offer an attractive platform to better understand and characterize defects in the ability of neutrophils to chemotax and the mechanism of neutrophil movement in response to stimuli presented as chemical gradients. LAP is the best-characterized periodontal disease showing impaired neutrophil function. Whether there is cross-correlation between neutrophil abnormalities in patients with chronic periodontitis and patients with LAP remains to be elucidated.

We have previously reported that peripheral blood neutrophils from patients with chronic periodontitis are both hyperactive and hyperreactive with respect to ROS generation (Matthews et al. 2007a,b) and that potential stimulants of these responses within plasma include GM-CSF, CXCL8 and interferon-α (IFN-α) (Dias et al. 2011). IFN-α is also capable of priming neutrophils within the circulation of patients with periodontitis and demonstrates elevated plasma levels, consistent with reported IFN-α responsive gene expression profile in neutrophils from patients with periodontitis (Wright et al. 2008). However, an element of ROS hyperactivity in periodontitis neutrophils appears to be constitutive (an innate property), and this may be due to an altered intracellular redox state in neutrophils from patients with periodontitis (Dias et al. 2013). Additionally, *Porphyromonas gingivalis*-derived gingipains can cleave CXCL8, potentially impacting upon ROS production and chemotaxis (Dias et al. 2008). This may be one plausible explanation why, in the present study, we observed a normalization of patient neutrophil responses to CXCL8 following successful treatment, but not for fMLP. Of the various neutrophil chemoattractants reported in the literature, a number of the host-derived chemoattractants demonstrated a similar pattern of activity to IL-8. We found similar results to IL-8 for GM-CSF and macrophage inhibitory protein 1alpha (MIP1α) (data not shown) whose receptors are all G-protein-coupled receptor linked. The enhanced ROS generation we have reported previously and the defective chemotactic accuracy observed in this study of patients with chronic periodontitis have also been shown in individuals with other inflammatory-driven diseases, such as rheumatoid arthritis (RA) (Biemond et al. 1986, Miesel et al. 1996, Bostan et al. 2002, Cedergren et al. 2007) and chronic obstructive pulmonary disease (COPD) (Rahman et al. 1997, Yoshikawa et al. 2007; Sapey at al. 2011).

Information on the mechanisms underlying altered neutrophil chemotaxis in LAP neutrophils may help in understanding aberrant chemotaxis in chronic periodontitis neutrophils. Several studies have reported a diminished capacity of labelled fMLP to bind to neutrophils in individuals with LAP, indicating a reduction in the number of peptide binding sites on the surface of LAP neutrophils, though the receptors themselves appear to be functional (Van Dyke et al. 1981, 1986). This would explain the diminished responsiveness of these neutrophils to a chemoattractive gradient. Defective LAP neutrophils also express lower levels of the surface glycoprotein gp110 (ADRM1 or hRpn13) (Van Dyke et al. 1987, 1990); the significance of this receptor was demonstrated by use of the monoclonal antibody to GP110 called NCD-1, which diminished chemotaxis in healthy neutrophils when exposed to fMLP (Cotter et al. 1981). LAP-defective neutrophils also have a reduced expression of CD38, another receptor for the chemoattractant fMLP (Fujita et al. 2005).

Chemoattractant–receptor binding results in the activation of numerous signalling pathways, including PI3K, which in turn mediates the activity of phosphoinositide dependent kinase 1 (PDK1), which has been shown to be an essential regulator of neutrophil chemotaxis (Fig. 1). A study by Yagi et al. (2009) demonstrated that neutrophils from LAP patients had reduced PDK-1 expression and activity. Proteomic analysis of defective LAP neutrophils revealed upregulation of four proteins, of which the actin binding protein caldesmon was considered the most significant and it was suggested that an increase in expression of this protein within the cell may suppress motility by stabilizing actin filaments (Mizuno et al. 2011). Other findings in defective neutrophils include reduced influx of extracellular calcium, lower calcium-dependent PKC activity in unstimulated defective LAP neutrophils, accumulation of diacylglycerol (DAG) and reduced DAG kinase activity in defective LAP neutrophils compared to healthy matched controls (Agarwal et al. 1989, Tyagi et al. 1992, Kurihara et al. 1993). DAG is an activator of PKC, functioning as a second messenger in a variety of cell functions including superoxide production and chemotaxis (Nishizuka 1986, Harvath et al. 1987, Lambeth 1988). Elevated DAG levels in abnormal LAP neutrophils support these neutrophils being kept in a primed state for activation. The actin polymerization and depolymerization in LAP individuals was found to be normal, supporting studies that show defects in the chemotaxis signalling cascade (Champagne et al. 1998). There is limited data in the literature on chemotaxis in neutrophils derived from patients with CP and all the above factors need to be analysed in patients with chronic periodontitis, such that we can further understand the altered processes in neutrophil chemotaxis in this disease. Indeed, it is interesting to speculate that such neutrophil deficiencies may represent a common mechanistic link between the different clinical definitions of periodontitis (e.g. LAP and CP).

Neutrophils are one of the key cells involved in protecting the host from bacterial challenge. Disruption to neutrophil functions such as chemotaxis may pre-dispose the individual to further infection and inflammation, exacerbating disease pathogenesis. Impaired neutrophil function impacts strongly on the ability of an individual to cope with microbial challenge as in periodontitis. Reduced neutrophil chemotactic accuracy and velocity may affect the transit time of neutrophils that have exited the circulation to reach the site of infection, potentially allowing bacteria to establish themselves in the periodontal tissues with greater potency. Collateral tissue damage may also arise as a result of prolonged tissue transit times secondary to defective chemotaxis, thus contributing to the chronic inflammatory burden.

In conclusion, we have demonstrated for the first time that neutrophils from patients with chronic periodontitis have reduced speed, velocity and chemotactic accuracy. Anti-infective treatment partially restores velocity and speed of neutrophil movement towards CXCL8 to control levels following periodontal therapy, but not for fMLP. Coupled with our previous knowledge that neutrophils from patients with periodontitis are both hyperactive and hyperreactive with respect to extracellular ROS production, which may drive increased tissue destruction, these findings may help to understand the potential role of dysfunctional neutrophils in the pathogenesis of periodontitis.

## Clinical Relevance

*Scientific rationale for the study*: Neutrophils are the predominant cell in the periodontal tissues. They are essential for clearance of bacteria but can also elicit host damage by inaccurately targeted responses. Movement of neutrophils to the epithelial surface requires degradation of tissue. Inaccurate movement can exacerbate tissue destruction.

*Principal findings*: Neutrophils from patients with chronic periodontitis display aberrant neutrophil movement that is only partially restored to healthy levels upon treatment.

*Practical implications*: Understanding sustained atypical neutrophil behaviour in periodontitis may help in the development of new therapeutic approaches.
